# Potent immunomodulation and angiogenic effects of mesenchymal stem cells versus cardiomyocytes derived from pluripotent stem cells for treatment of heart failure

**DOI:** 10.1186/s13287-019-1183-3

**Published:** 2019-03-07

**Authors:** Songyan Liao, Yuelin Zhang, Sherwin Ting, Zhe Zhen, Fan Luo, Ziyi Zhu, Yu Jiang, Sijia Sun, Wing-Hon Lai, Qizhou Lian, Hung-Fat Tse

**Affiliations:** 1 Cardiology Division, Department of Medicine, Queen Mary Hospital, The University of Hong Kong, Rm 1928, Block K, Hong Kong SAR, China; 2 Shenzhen Institutes of Research and Innovation, The University of Hong Kong, Shenzhen, China; 3 Department of Emergency, Guangdong Academy of Medical Sciences, Guangzhou, Guangdong China; 4 Bioprocessing Technology Institute, A*STAR (Agency for Science, Technology and Research), Singapore, 138668 Singapore; 5 Research Center of Heart, Brain, Hormone and Healthy Aging, Li Ka Shing Faculty of Medicine, The University of Hong Kong, Hong Kong SAR, China; 6 Hong Kong-Guangdong Joint Laboratory on Stem Cell and Regenerative Medicine, The University of Hong Kong and Guangzhou Institutes of Biomedicine and Health, Hong Kong SAR, China

**Keywords:** Pluripotent stem cells, Mesenchymal stem cell, Cardiomyocytes, Heart failure

## Abstract

**Background:**

Optimal cell type as cell-based therapies for heart failure (HF) remains unclear. We sought to compare the safety and efficacy of direct intramyocardial transplantation of human embryonic stem cell-derived cardiomyocytes (hESC-CMs) and human induced pluripotent stem cell-derived mesenchymal stem cells (hiPSC-MSCs) in a porcine model of HF.

**Methods:**

Eight weeks after induction of HF with myocardial infarction (MI) and rapid pacing, animals with impaired left ventricular ejection fraction (LVEF) were randomly assigned to receive direct intramyocardial injection of saline (MI group), 2 × 10^8^ hESC-CMs (hESC-CM group), or 2 × 10^8^ hiPSC-MSCs (hiPSC-MSC group). The hearts were harvested for immunohistochemical evaluation after serial echocardiography and hemodynamic evaluation and ventricular tachyarrhythmia (VT) induction by in vivo programmed electrical stimulation.

**Results:**

At 8 weeks post-transplantation, LVEF, left ventricular maximal positive pressure derivative, and end systolic pressure-volume relationship were significantly higher in the hiPSC-MSC group but not in the hESC-CM group compared with the MI group. The incidence of early spontaneous ventricular tachyarrhythmia (VT) episodes was higher in the hESC-CM group but the incidence of inducible VT was similar among the different groups. Histological examination showed no tumor formation but hiPSC-MSCs exhibited a stronger survival capacity by activating regulatory T cells and reducing the inflammatory cells. In vitro study showed that hiPSC-MSCs were insensitive to pro-inflammatory interferon-gamma-induced human leukocyte antigen class II expression compared with hESC-CMs. Moreover, hiPSC-MSCs also significantly enhanced angiogenesis compared with other groups via increasing expression of distinct angiogenic factors.

**Conclusions:**

Our results demonstrate that transplantation of hiPSC-MSCs is safe and does not increase proarrhythmia or tumor formation and superior to hESC-CMs for the improvement of cardiac function in HF. This is due to their immunomodulation that improves in vivo survival and enhanced angiogenesis via paracrine effects.

**Electronic supplementary material:**

The online version of this article (10.1186/s13287-019-1183-3) contains supplementary material, which is available to authorized users.

## Background

Heart failure (HF) following myocardial infarction (MI) remains one of the leading causes of mortality and morbidity worldwide [1]. Despite the use of evidence-based medical therapies, including coronary revascularization, angiotensin-converting enzyme, and β-blocker drugs, a significant proportion of patients develops pathological left ventricular (LV) remodeling and progressive HF due to irreversible loss of cardiomyocytes following MI [2]. As a result, cell-based therapeutics have been explored as a potential approach to replenish the lost cardiomyocytes and improve LV function in HF following MI [3]. The majority of early clinical trials of cell-based therapy for the treatment of MI were conducted using autologous bone marrow (BM) mononuclear cells. Unfortunately, recent clinical studies [4–6] and meta-analysis [7, 8] have failed to demonstrate any consistent improvement in LV function or infarct size. One of the potential reasons for the discordance in clinical efficacy between studies is the poor survival of transplanted cells and the innate diversity of cell number and populations yielded from BM stem cells in different patients [9]. In this regard, pluripotent stem cells, including cells derived from human embryonic stem cells (hESCs) and induced pluripotent stem cells (hiPSCs) that are capable of high volume quality-controlled production, should provide more predictable therapeutic effects and “off-the-shelf” usage without the need for preparation.

Recent preclinical [10–12] and clinical studies [13] have demonstrated the feasibility of generating hESC-derived cardiomyocytes (hESC-CMs) or induced pluripotent stem cell-derived cardiomyocytes (hiPSC-CMs) that are of sufficient quality for transplantation. Moreover, successful engraftment and electrical integration with native host myocardium have been achieved with transplantation of these pluripotent stem cell-derived cardiomyocytes in non-human primate models of MI. Nevertheless, inconsistent improvement in LV function was observed after transplantation [10–12]. MSC is another cell source and has been widely used for treating MI and HF in preclinical and clinical studies. Previous studies have shown that the improvements in infarct size and heart function are observed when MSCs are transplanted into hearts [14–16]. We have recently demonstrated that human MSCs derived from hiPSCs (hiPSC-MSCs) show better proliferative capacity, survival, and therapeutic efficacy for tissue repair than BM-derived MSCs (BM-MSCs) [17, 18]. There has been no direct comparison of the therapeutic potential of pluripotent stem cell-derived CMs with that of MSC transplantation for cardiac repair in HF. Accordingly, we compared the safety and efficacy of hESC-CM versus hiPSC-MSC transplantation in a large animal model of HF induced by MI and rapid pacing.

## Methods

An extended Materials and Methods are available in Additional file 1.

### Cell culture

In this study, a total of three batches of IMR90-iPSC-MSCs and four batches of HES-3-ESC-CMs were used for cell transplantation. The culture and differentiation of MSCs derived from human IMR90-iPSC lines (WiCell Research Institute, Madison, WI, USA) [18, 19] and CMs derived from HES-3-ESC lines (ES Cell International, Singapore) [20] have been described previously. Flow cytometry analysis or surface antigen profiling of iPSC-MSCs (Additional file 1: Figure S1) and anti-cardiac troponin T (cTnT, 1:200; Thermo Scientific) of hESC-CMs (Additional file 1: Figure S2) showed consistent quality of differentiated cells in each batch before transplantation. Cellular electrophysiological properties of the differentiated cardiomyocytes were measured by a patch clamping technique on action potential (Additional file 1: Figure S3). In this study, another two more cell lines (Lee NL-iPSC-MSC generated from our lab [17] and H-7-hESC-CM acquired from WiCell Research Institute, Madison, WI, USA) were used for comparing in a cytokine array and western blot.

### Animal model

Female farm pigs weighing 35–40 kg (9–12 months old) were used for this study. A large animal model of HF induced by MI and rapid pacing was created as described previously [21–23]. In brief, all animals were anesthetized with zoletil (tiletamine+zolezepam, 2–7 mg/kg, IM) and xylazine (0.5–1 mg/kg, IM). Endotracheal intubation was performed, and anesthesia was maintained with isoflurane (1.5 to 2.0%, continuous inhalation) and oxygen (0.5–1.5 L/min, inhalation) while the animals were mechanically ventilated. Coronary angiography was performed through a 6F JR4 guiding catheter (Cordis Corp, Miami, FL, USA) via the right femoral artery. The left circumflex coronary artery distal to the first obtuse marginal branch was occluded with balloon inflation, and 700 μm microspheres were injected to precipitate MI (Additional file 1: Figure S4). A pacemaker (St. Jude Medical) was implanted into the pig’s neck with a pacemaker lead connected to the right ventricle. During the procedure, LV pressure was monitored to ensure at least a 30% reduction in +dP/dt following MI. A surface electrocardiogram and arterial blood pressure were continuously monitored throughout the procedure. All animals received amiodarone (300 mg intravenously over 1 h) and lidocaine (100 mg intravenous bolus) before infarction to prevent or treat ventricular arrhythmias. In those animals who survived 4 weeks post-MI, an additional 4 weeks of rapid ventricular pacing (150 bpm) was applied to induce HF. The pacemaker was then reprogrammed at 8 weeks to backup VVI mode (35 bpm). At the end of the experiment, the animals will be euthanized by an overdose of Dorminal (pentobarbital sodium, 100 mg/kg, IV). All animal experiments were performed in accordance with the Guide for the Care and Use of Laboratory Animals published by the US National Institutes of Health and regulations of the University of Hong Kong, and the protocol was approved by the Committee on the Use of Live Animals in Teaching and Research (CULTAR) at the University of Hong Kong.

### Cell transplantation

Eight weeks after the induction of HF, animals that developed significant impairment of left ventricular ejection fraction (LVEF) which at least a 40% reduction of normal LVEF were randomly assigned to receive direct intramyocardial injection of saline (MI group), 2 × 10^8^ hESC-CMs (hESC-CM group), or 2 × 10^8^ hiPSC-MSCs (hiPSC-MSC group). All animals were anesthetized with tiletamine and zolezepam (zoletil, 20 mg/kg, IM) as mentioned above and underwent left thoracotomy and pericardiotomy to expose the infarcted lateral wall. A total of 10–12 injections (0.2 ml per injection) were performed around the infarct area (Additional file 1: Figure S5). All animals received immunosuppression with oral steroid (40 mg/day orally) and cyclosporine (200 mg/day orally) 3 days before transplantation and continued for 8 weeks afterwards. In addition, all animals were treated with conventional pharmacological therapies for HF with daily oral metoprolol succinate (25 mg) plus ramipril (2.5 mg).

### Echocardiographic and invasive hemodynamic assessment

All animals were anesthetized with tiletamine and zolezepam (zoletil, 20 mg/kg, IM) as mentioned above. Details of the echocardiographic and invasive hemodynamic assessment are described in Materials and Methods in the online-only Additional file 1.

### Intracardiac programmed electrical stimulation

Programmed electrical stimulation was performed to assess the inducibility of ventricular tachyarrhythmia (VT) after cell therapy before sacrificing the animal. Full details are described in Materials and Methods in the online-only Additional file 1*.*

### Statistical analysis

All data are expressed as mean ± SEM, and analysis was performed using SPSS software (SPSS, Inc., Chicago, IL, USA). All the analysis of the echocardiographic, invasive hemodynamic, and histological measurements was done in a blinded fashion. The Student *t* test was used to compare two groups. Comparison of variables between multiple groups was performed using repeated measures two-way ANOVA and one-way ANOVA with Bonferroni post hoc test. A *P* value ≤ 0.05 was considered statistically significant.

## Results

**Fig. 1:**
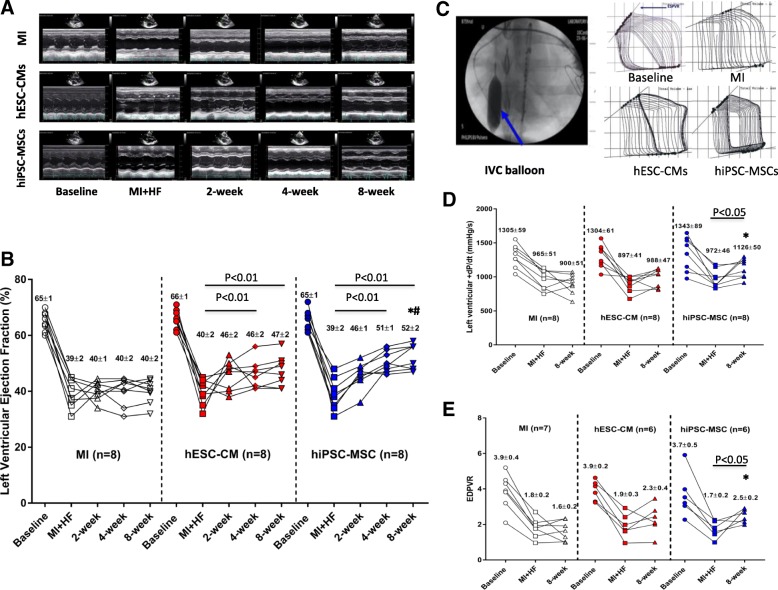
Improvement of left ventricular (LV) function after cell transplantation. **a** LV M-mode echocardiogram image at baseline, heart failure (HF), and 2, 4, and 8 weeks after cell transplantation. **b** Serial echocardiographic measurements of left ventricular ejection fraction (LVEF) in myocardial infarction (MI) group(*n* = 8), human embryonic stem cell-derived cardiomyocyte (hESC-CM) group (*n* = 8) or human induced pluripotent stem cell-derived mesenchymal stem cell (hiPSC-MSC) group (*n* = 8) at different time points. LVEF was significantly increased in the hESC-CM and hiPSC-MSC groups at 4 and 8 weeks after transplantation than during HF, but not in the MI group. Moreover, LVEF was significantly higher in the hiPSC-MSC group than in the MI group (^#^*P* < 0.01 vs. MI using repeated measures two-way ANOVA with Tukey’s post hoc test) and hESC-CM group (*P < 0.05 vs. hESC-CM using repeated measures two-way ANOVA with Tukey’s post hoc test) at 8 weeks after transplantation. **c**–**e** Maximal left ventricular positive pressure derivative (+dP/dt) and end systolic pressure-volume relationship (ESPVR) was measured by invasive hemodynamic assessment of pressure-volume (PV) loops. ESPVR was measured during occlusion of the inferior vena cava (IVC) by balloon inflation (blue arrow). At 8 weeks, both left ventricular +dP/dt and ESPVR in the hiPSC-MSC group were significantly increased compared with during HF and were significantly higher than the MI group (*P < 0.05 vs. MI using repeated measures two-way ANOVA with Tukey’s post hoc test)

A total of 28 pigs with MI were randomized to receive saline (MI group, *n* = 9), hESC-CMs (*n* = 10), or hiPSC-MSCs (*n* = 9). There were no significant differences in the LVEF measured by echocardiogram between the MI, hESC-CM, and hiPSC-MSC groups, respectively (Fig. 1a, b; 38.7% vs. 39.3% and 37.5%, *P* > 0.1 before cell transplantation). One animal each from the MI group and hiPSC-MSC group and two from the hESC-CM group died within 3 days of the transplantation procedure. As a result, eight animals from each group completed the study protocol.

### Improvement of LV function after cell transplantation

Serial echocardiographic examination showed that LVEF was significantly decreased after MI in groups from 65.0 ± 1.49%, 65.88 ± 1.4%, and 65.13 ± 1.3% at baseline to 39.44 ± 2.15%, 39.29 ± 2.26%, and 39.88 ± 2.49% at 8 weeks after induction of HF (Fig. 1a, b; *P < 0.01*). There were no significant differences in LVEF among the three groups at baseline and during HF (Fig. 1b; *P* > 0.1). LVEF was significantly increased in hESC-CM and hiPSC-MSC groups at 4 and 8 weeks after transplantation compared with during HF, but not in the MI group (Fig. 1b; *P < 0.01*). More importantly, LVEF was also significantly higher in the hiPSC-MSC group than hESC-CM group at 8 weeks after transplantation (Fig. 1b; *P <* 0.05). Detailed echocardiographic parameters are shown in Additional file 1: Table S1. There are no significant differences in left ventricular end-diastolic dimension, left atrial dimension, and E/A ratio among the three groups. However, the left ventricular end-systolic dimension was significantly decreased in the hiPSC-MSC group at 4 and 8 weeks after transplantation compared with during HF (*P* < 0.05).

Invasive hemodynamic assessment of pressure-volume loop was performed to measure LV maximal positive pressure derivative (+dP/dt) and the end systolic pressure-volume relationship (ESPVR) (Fig. 1c). The +dP/dt (Fig. 1d) and ESPVR (Fig. 1) were similar among the three groups at baseline and during HF. Compared with the MI group, the +dP/dt and ESPVR were significantly higher in the hiPSC-MSC group (Fig. 1d, e; *P <* 0.05), but not in the hESC-CM group at 8 weeks after transplantation (Fig. 1d, e; *P* > 0.05). Moreover, the +dP/dt and ESPVR were significantly higher in the hiPSC-MSC group at 8 weeks after transplantation than during HF (Fig. 1d, e; *P <* 0.05), but not in the MI or hESC-CM groups (Fig. 1d, e; *P* > 0.05). Taken together, our results show that transplantation of hiPSC-MSCs is superior to hESC-CMs for the improvement of LV function in HF.

To determine whether cell transplantation increases arrhythmogenic complications, telemetry monitoring from the pacemaker and electrophysiological study were performed to assess the incidence of spontaneous and inducible ventricular arrhythmias 8 weeks after cell transplantation, respectively. The incidence of spontaneous non-sustained ventricular tachyarrhythmia (rate > 180 bpm and > 12 beats) was higher in the hESC-CM group (75%) than the hiPSC-MSC group (12.5%) and MI group (25%, Additional file 1: Figure S6; *P <* 0.05). Nevertheless, the incidence of inducible sustained VT was similar among the three groups (62.5% in the hiPSC-MSC group, 75% in the hESC-CM group, and 75% in the MI group, Additional file 1: Figure S6; *P* > 0.05).

### Infarct size changes after cell transplantation

**Fig. 2:**
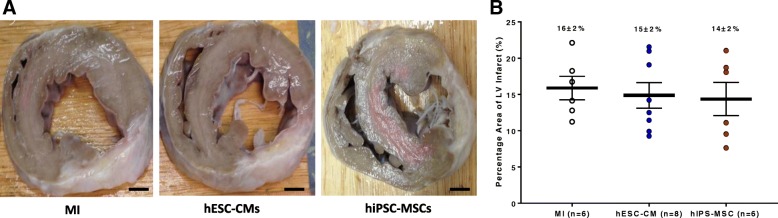
Infarct area after cell transplantation. **a** LV transverse direction samples sectioned at 1 cm thickness in each animal that contained infarcted myocardium(bar = 1 cm). **b** There was no significant difference in infarct area among the three groups at 8 weeks after cell transplantation

The average LV wall thickness at the infarct region was measured from serial 1 cm thickness section samples (ranged from 5 to 7 sections) in each animal (Fig. 2a). There was no significant difference in the percentage infarcted area normalized to body weight in the hiPSC-MSC group and hESC-CM group compared with the MI group (Fig. 2b; *P* > 0.05). These results suggest that hESC-CM or hiPSC-MSC transplantation does not lead to regeneration of the infarcted myocardium. Furthermore, we examined the myocardial samples that comprise approximately 1 cm^2^ piece at five random fields in each section. The specimens were embedded in paraffin and sectioned into 5 μm slices for detailed histological examination after H&E staining. A similar method was also used in other non-cardiac organs including the lung, liver, kidney, and duodenum. We did not observe any tumor formation at the injection areas as well as the other sites over the myocardium or other organs. The representative histological sections from different organs are now shown in Additional file 1: Figure S7.

### Neovascularization after cell transplantation

**Fig. 3:**
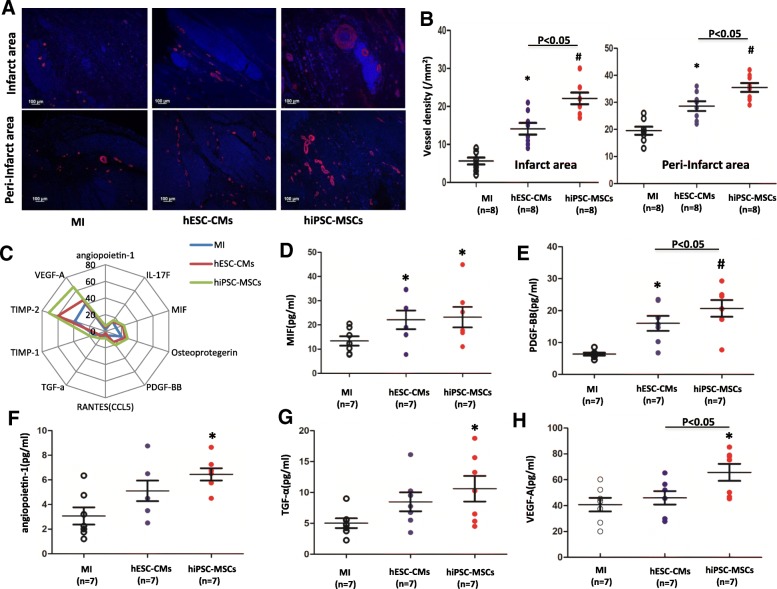
Neovascularization after cell transplantation. **a** Anti-SMA staining for capillary density (red color) per × 10 section of peri-infarct regions at 8 weeks after transplantation in three groups (bar = 100 μm). **b** There was significant enhancement of capillary density 8 weeks after hESC-CM and hiPSC-MSC transplantation compared with the MI group (*P < 0.05 vs. MI, ^#^*P* < 0.01 vs. MI using one-way ANOVA with Bonferroni post hoc test); moreover, hiPSC-MSC significantly increased capillary density compared with the hESC-CM group (*P* < 0.05 vs. hESC-CMs using one-way ANOVA with Bonferroni post hoc test) in both infarct area and peri-infarct area. **c** Angiogenic cytokines in heart tissue at the peri-infarct area were measured using the porcine cytokine array kit. **d**–**h**. Macrophage migration inhibitory factor (MIF), platelet-derived growth factor subunit B (PDGF-BB), angiopoietin-1, transforming growth factor alpha (TGF-α), and vascular endothelial growth factor A (VEGF-A) were increased significantly in the hiPSC-MSC group compared with MI group (*P < 0.05 vs. MI, ^#^*P* < 0.01 vs. MI using one-way ANOVA with Bonferroni post hoc test)

To determine whether increased vessel growth could contribute to the functional improvements observed in hiPSC-MSC-treated hearts, we used alpha smooth muscle actin (α-SMA) antibody to stain the vessel formation at the infarct and peri-infarct area 8 weeks after cell transplantation (Fig. 3a). Compared with the MI group and hESC-CM group, the vessel density was significantly higher in the hiPSC-MSC group in both the infarct area and peri-infarct area (Fig. 3b; *P <* 0.01).

As shown in Fig. 3c, angiogenic cytokines in heart tissue at the peri-infarct area were measured using a porcine cytokine array kit. Macrophage migration inhibitory factor (MIF) and platelet-derived growth factor subunit B (PDGF-BB) were significantly higher in hiPSC-MSC and hESC-CM groups compared with the MI group (Fig. 3d; *P <* 0.05). Nonetheless compared with the MI group, angiopoietin-1, transforming growth factor alpha (TGF-α), and vascular endothelial growth factor A (VEGF-A) were markedly increased only in the hiPSC-MSC group, not in the hESC-CM group (Fig. 3f–h; *P* < 0.05). These results show that hiPSC-MSCs can stimulate angiogenesis by upregulating the myocardial expression of some angiogenic cytokines after transplantation.

#### Secretome of hiPSC-MSCs promotes neovascularization in vitro

**Fig. 4:**
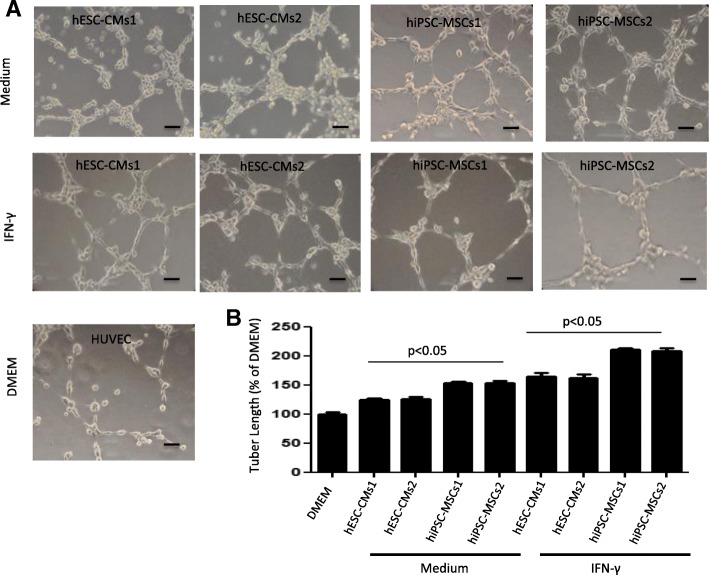
Secretome of hESC-CMs and hiPSC-MSCs promotes neovascularization in vitro. **a** Tube formation assay was performed with human umbilical vein endothelial cells (HUVECs) treated with Dulbecco’s modified Eagle’s medium (DMEM) or the conditioned medium (CdM) from hESC-CMs (two cell lines) and hiPSC-MSCs (two cell lines) (bar = 50 μm). **b** The tube length was analyzed among the different groups. Compared with DMEM, tube length was increased in HUVECs cultured with CdM from hiPSC-MSCs and hESC-CMs as well as dramatically increased by interferon-γ (IFN-γ) stimulation. Moreover, the tube length was much longer in CdM from hiPSC-MSCs than CdM from hESC-CMs with and without IFN-γ stimulation (*P* < 0.05 using one-way ANOVA with Bonferroni post hoc test)

To examine the angiogenic capacity of the secretome of hiPSC-MSCs and hESC-CMs, we cultured human umbilical vein endothelial cells (HUVECs) with Dulbecco’s modified Eagle’s medium (DMEM) or the conditioned medium (CdM) derived from hiPSC-MSCs and hESC-CMs on Matrigel in the tube formation assay. The tube length was notably increased in HUVECs cultured with CdM from hiPSC-MSCs and hESC-CMs compared with DMEM. Tube length was also much longer in CdM from hiPSC-MSCs than CdM from hESC-CMs, indicating that hiPSC-MSCs have a stronger angiogenic capacity than hESC-CMs (Fig. 4a, b; *P <*0.05). The angiogenic capacity of hiPSC-MSCs and hESC-CMs was further augmented by stimulation with interferon-γ (IFN-γ). Tube length was dramatically increased when HUVECs were cultured with CdM from hiPSC-MSCs and hESC-CMs with IFN-γ stimulation compared with no IFN-γ stimulation (Fig. 4a, b). Notably, the tube length of HUVECs treated with CdM from hiPSC-MSCs with IFN-γ stimulation was much longer than in those treated with CdM from hESC-CMs with IFN-γ stimulation (Fig. 4a, b; *P* < 0.05).

#### Cytokine assays of secretome from hiPSC-MSCs and hESC-CMs

**Fig. 5:**
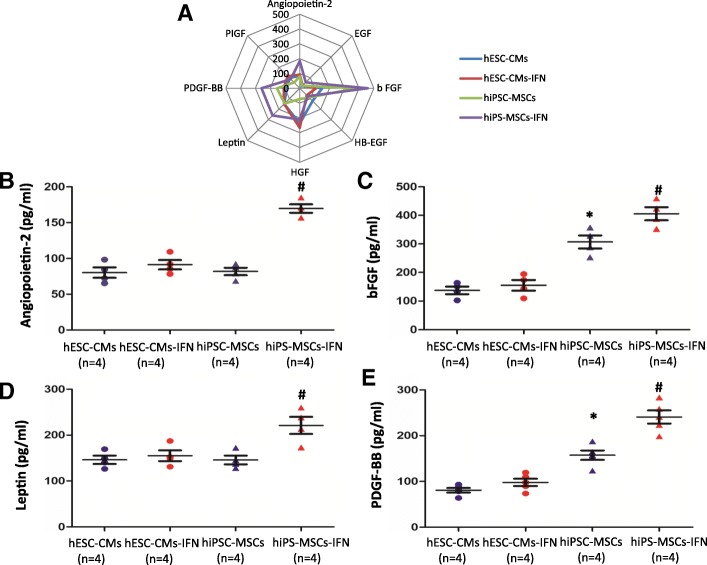
Cytokine assays of secretome from hESC-CMs or hiPSC-MSCs. **a** Angiogenic cytokines in CdM from hiPSC-MSCs (two cell lines) and hESC-CMs (two cell lines) with or without IFN-γ stimulation were measured by the cytokine assays. **b**–**e** The expression of angiopoietin-2, basic fibroblast growth factor (bFGF), leptin, and PDGF-BB from hiPSC-MSCs was much higher than from hESC-CMs with IFN-γ stimulation (*P < 0.05 vs. hESC-CMs, ^#^*P* < 0.01 vs. hESC-CMs-IFN using one-way ANOVA with Bonferroni post hoc test)

To further investigate the angiogenic capacity of hiPSC-MSCs and hESC-CMs, we collected the CdM from hiPSC-MSCs and hESC-CMs with or without IFN-γ stimulation and then performed cytokine assays (Fig. 5a). Angiopoietin-2, basic fibroblast growth factor (bFGF), leptin, and PDGF-BB were increased in hiPSC-MSCs with IFN-γ stimulation compared with hESC-CMs with IFN-γ stimulation (Fig. 5b–e; *P <* 0.05) measured using a human cytokine array kit for angiogenesis. This indicated that hiPSC-MSCs possess a stronger angiogenic capacity than hESC-CMs responsive to IFN-γ stimulation by regulating the expression of several angiogenic cytokines.

### Cell survival after transplantation

**Fig. 6:**
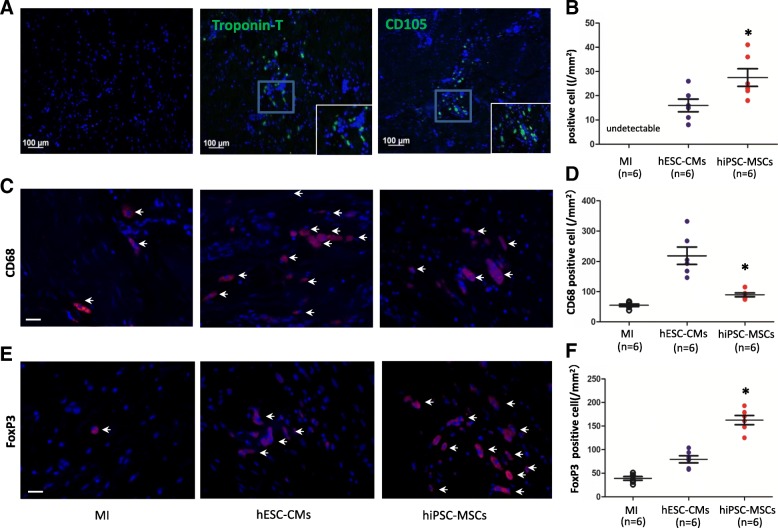
Cell survival after hESC-CM or hiPSC-MSC transplantation. **a** Troponin-T and CD105 staining for cell survival of peri-infarct regions 8 weeks after transplantation in the three groups (red color). **b.** The cell survival of hiPSC-MSCs was significantly higher than that of hESC-CMs (*n* = 6 in each group, *P < 0.05 vs. hESC-CMs using the Student *t* test). **c** Macrophage marker CD68 immunostaining for macrophage expression of peri-infarct regions 8 weeks after transplantation in the three groups (red color, bar = 20 μm). **d** hiPSC-MSCs reduced the number of macrophages compared with hESC-CMs (*n* = 6 in each group, *P < 0.05 vs. hESC-CMs using one-way ANOVA with Bonferroni post hoc test). **e** Anti-FOXP3 antibody immunostaining for regulatory T cell expression of peri-infarct regions 8 weeks after transplantation in the three groups (red color, bar = 20 μm). **f** hiPSC-MSCs also increased the number of regulatory T cells compared with hESC-CMs (*n* = 6 in each group, *P < 0.05 vs. hESC-CMs using one-way ANOVA with Bonferroni post hoc test). The total cell nucleus in all groups was stained with DAPI (blue color)

The survival of transplanted hESC-CMs and hiPSC-MSCs in peri-infarct regions was detected by immunostaining for anti-cardiac Troponin-T or CD105 at 8 weeks after transplantation (Fig. 6a). There is no cell survival around the inject site in the infarct area, but the percentage survival of hiPSC-MSCs was much higher than that of hESC-CMs in the peri-infarct area (Fig. 6b; *P <* 0.05). Furthermore, compared with the hESC-CM group, the number of macrophages was lower (Fig. 6c, d; *P <* 0.05) in the hiPSC-MSC group, measured by immunostaining of macrophage marker CD68. The number of regulatory T cells (Fig. 6e, f; *P <* 0.05) was higher, measured by anti-FOXP3 antibody in the myocardium. These results suggest that higher hiPSC-MSC survival after transplantation is associated with a decline in myocardial inflammation, consistent with a previous report of the immunomodulatory effect of MSCs [24].

#### Distinct expression of human leukocyte antigen between hiPSC-MSCs and hESC-CMs

**Fig. 7:**
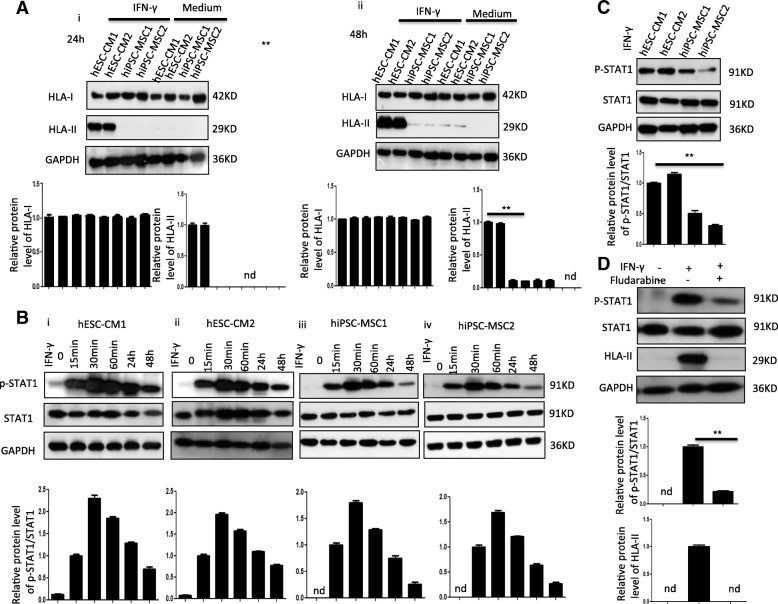
Distinct expression of human leukocyte antigen (HLA) between hESC-CMs and hiPSC-MSCs. **a** The expression of HLA class I (HLA-I) and class II (HLA-II) in hiPSC-MSCs (two cell lines) and hESC-CMs (two cell lines) after 1 (**i**) and 2 days (**ii**) in the presence or absence of IFN-γ. HLA-II was not expressed in hiPSC-MSCs but weakly expressed in hESC-CMs. Expression of HLA-II was significantly increased in hESC-CMs but not in hiPSC-MSCs after IFN-γ stimulation for 24 h and 48 h (**i**, **ii**). **b** The expression of signal transducer and activator of transcription 1 (P-STAT1) at different time points after IFN-γ stimulation was detected in hESC-CMs (**i**, **ii**) and hiPSC-MSCs (**iii**, **iv**). **c** The hiPSC-MSCs exhibited lower levels of P-STAT1 2 days after IFN-γ stimulation compared with hESC-CMs. **d** The expression of P-STAT1 and HLA-II in hESC-CMs was significantly enhanced in response to IFN-γ stimulation and reduced when cells received fludarabine treatment (*P < 0.05 vs. hESC-CMs using one-way ANOVA with Bonferroni post hoc test)

The other potential mechanism for a superior survival rate of hiPSC-MSCs compared with hESC-CM post-transplantation is their difference in allogenic response that is regulated by human leukocyte antigen (HLA) class I (HLA-I) and class II (HLA-II) expression. A lower level of HLA-II reduces the alloreactivity risk [25]. Accordingly, we measured the expression of HLA-I and HLA-II in hiPSC-MSCs and hESC-CMs. Western blot results showed that under normal conditions, both iPSC-MSCs and hESC-CMs express a high level of HLA-I. Nonetheless, HLA-II was not expressed in iPSC-MSCs but expressed in hESC-CMs (Fig. 7**a** (**i**, **ii**)). In contrast, after IFN-γ stimulation for 24 h and 48 h, the expression of HLA-II was significantly increased in hESC-CMs but not in iPSC-MSCs, suggesting that hiPSC-MSCs have a higher level of immune privilege than hESC-CMs. This may account for the higher survival rate of hiPSC-MSCs after transplantation in the infarcted heart compared with hESC-CMs. There was no change to the expression of HLA-I in hiPSC-MSCs or hESC-CMs in response to IFN-γ stimulation.

Accumulating evidence has demonstrated that phosphorylation of signal transducer and activator of transcription 1 (P-STAT1) is induced by pro-inflammation and activates class II major histocompatibility complex transactivator (CIITA) to stimulate the transcription of major histocompatibility complex II (MHC-II) molecules [25, 26]. We therefore further examined the STAT1 signaling pathway in iPSC-MSCs and hESC-CMs under IFN-γ challenge. The P-STAT1 was dramatically increased from 15 min after IFN-γ stimulation and peaked at 30–60 min and gradually decreased at 60 min, then was finally maintained at a high level at 48 h in both hiPSC-MSCs and hESC-CMs (Fig. 7b). Furthermore, at 48 h of IFN-γ stimulation, the expression of P-STAT1 was much higher in hESC-CMs than hiPSC-MSCs (Fig. 7c). To further verify that P-STAT1 regulates the expression of MHC-II, we used fludarabine to inhibit P-STAT1. As shown in Fig. 7d, expression of P-STAT1 and HLA-II in hESC-CMs was significantly enhanced in response to IFN-γ stimulation. Nonetheless, expression of HLA-II was remarkably reduced when cells were treated with fludarabine, indicating that P-STAT1 mediates the transcription of MHC-II molecules (Fig. 7d).

## Discussion

The optimal cell source for the treatment of post-MI HF remains unclear [3, 9]. Compared with somatic stem cells such as bone marrow-derived MSCs, pluripotent stem cell-derived cell sources, including cardiomyocytes and MSCs, should provide greater therapeutic benefits due to their higher proliferative capacity and ability to be used as an “off-the-shelf” product with batch to batch consistency. As mentioned above, recent non-human primate studies [10–12] have shown the feasibility of using hESC-CMs for cardiac repair. Pilot human trial has also demonstrated the safety and feasibility to derive clinical grade hESC line for cardiac progenitor cells for human transplant. Therefore, hESC-CM-based therapy is a potential feasible candidate for cell-based therapy for heart failure. On the other hand, our group has reported the generation of highly proliferative hiPSC-MSC for tissue repair. Indeed, clinical grade hiPCS-MSC has been developed by industries, for example, CymerusTM technology, for potential clinical uses. As a result, hiPCS-MSC will be soon available as another potential therapy for heart failure. In contrast, no clinical grade hESC-MSC is under development as therapeutic agent. Hence, we decided to compare the safety and efficacy of these two pluripotent stem cell-derived cell sources as a potential therapy for the treatment of heart failure in a clinically relevant large animal model of heart failure.

Our results demonstrated that (i) transplantation of hiPSC-MSCs or hESC-CMs is safe and does not increase proarrhythmic risk or tumor formation; (ii) hiPSC-MSC transplantation is superior to hESC-CMs for improvement of LV function in HF, attributed to their paracrine effects on the preservation of native cardiomyocytes and enhancement of neovascularization; and (iii) a greater therapeutic effect of hiPSC-MSCs than hESC-CMs is also related to their immunomodulatory effects via activation of regulatory T cells and immune privilege with lower expression of HLA-II to enhance cell survival after transplantation. These findings have important clinical implications in the development of stem cell-based therapy for the treatment of HF.

In this study, direct intramyocardial transplantation of hESC-CMs and iPSC-MSCs was performed after induction of HF. At 8 weeks follow-up, we observed significant improvement in LV function as determined by echocardiographic measurement of LVEF and invasive hemodynamic measurement of +dP/dt and ESPVR only after hiPSC-MSC transplantation, not after hESC-CM transplantation. Nonetheless, there was no evidence of myocardial regeneration following transplantation of hiPSC-MSCs or hESC-CMs since no changes to the infarct area or wall thickness were detected. Therefore, it is unlikely that the transplanted cells contributed directly to any improvement in LV function in this study. In contrast, recent studies in non-human primates showed that both allogeneic primate iPSC-CM [11] and hESC-CM [10, 12] transplantation resulted in significant remuscularization of the infarcted heart to provide sustained improvement in the LV function. There are several major differences in the experimental study design that might account for their conflicting results [10–12] with the current study. First, we induced MI by permanent coronary occlusion via embolization. However, the ischemia-reperfusion model was used in these studies to create MI [10–12]. The lack of sufficient blood supply in our study to the transplanted cells due to permanent coronary occlusion might contribute to their poor engraftment and survival after transplantation. Second, we performed cell transplantation at 8 weeks after MI rather than immediately [10, 11] or at 2 weeks [12] after MI. It is possible that the remuscularization of the infarcted heart by the transplanted cells is less optimal during the late healing phase after MI [27]. Third, primate to pig rather than primate to primate cell transplantation was performed in this study. Finally, the protocols for immunosuppression were different among these studies, and optimal immunosuppression regime in pig remains unclear.

On the other hand, hiPSC-MSC transplantation provided more potent protective effects on native cardiomyocytes as well as angiogenic effects than hESC-CM transplantation. Our data demonstrated that hiPSC-MSCs have a stronger protective effect than hESC-CMs in reducing cell hypertrophy and improving the survival of native cardiomyocytes at the peri-infarct zone following transplantation (Additional file 1: Figure S8). We strongly believe that these results provide evidences of preservation of native cardiomyocytes at the peri-infarct sites as one of the potential mechanisms for the improvement in cardiac function after cell transplantation observed in this study. Next, we further demonstrated that hiPSC-MSCs can enhance neovascularization both in vivo and in vitro by upregulating the expression of several angiogenic proteins such as MIF, PDGF-BB, TGF-α, VEGF-A, and angiopoietin-1. These beneficial effects might have contributed to the superior performance of hiPSC-MSCs.

One of the major hurdles to the clinical application of both adult stem cells and pluripotent stem cells are their poor survival and engraftment as well as lack of in vivo transdifferentiation following transplantation. Prior small animal studies have shown that only a very small proportion of MSCs [28], ESC-CMs [29], or even iPS cells [30] were observed in the infarcted myocardium after transplantation. Recent non-human primate studies also demonstrated that transplanted human MSC [14] or human ESC-derived cardiac progenitor cells [31] failed to remuscularize the infarcted myocardium with minimal engraftment but still consistently improved myocardial function. These results did provide evidences that human progenitor stem cells can enhance myocardial repair via their paracrine effects [32, 33] and other possible mechanisms such as mitochondrial transfer [17, 34]. In this study, we cannot find any cell survival around the inject site in the infarct area, but only can stain very few numbers of cells in the peri-infarct area. Moreover, the percentage cell survival of hiPSC-MSCs was significantly higher than that of hESC-CMs. hiPSC-MSC-treated hearts also tended to have fewer macrophages and more regulatory T cells with the expression of FOXP3, a specific marker for these cells. Regulatory T cells are thought to play a critical role in the control of T cell-mediated autoimmunity by suppressing the proliferation and cytokine production of other T cells [35]. These in vivo observations suggest that hiPSC-MSCs have potent immunomodulatory effects that can improve their survival after transplantation. Recent clinical studies [36] have also suggested that more potent immunomodulatory effects of allogenic MSCs might have contributed to better therapeutic effects than autologous MSCs for the treatment of HF.

Increasing evidence has demonstrated that HLA plays a critical role in the regulation of the allogeneic response of stem cell transplantation [37, 38]. Indeed, transplantation of the MHC-matched allogeneic iPSC-CMs from homozygous monkey survived for up to 12 weeks in the infarcted myocardium without evidence of immune rejection in non-human primate treated with methylprednisolone and tacrolimus only. On the other hand, transplantation of iPSC-CMs into MHC-mismatched non-human primate resulted in immune rejection with severe infiltration of T lymphocytes and minimal cell engraftment after transplantation [11]. MSCs are considered immune-privileged cells due to their low expression of HLA-I and lack of expression of HLA-II [39]. Consistent with previous studies [24, 40], we also were able to detect only HLA-I, not HLA-II, in two cell lines of iPSC-MSCs used in the current study. Nonetheless, expression of HLA-II was detected in hESC-CMs. HLA-II regulates immune recognition, and thus the absence of HLA-II should minimize immune rejection. The different expression level of HLA-II in hiPSC-MSCs and hESC-CMs in response to stimuli implies their different levels of immune privilege. After ischemia, the injured heart releases a lot of pro-inflammatory cytokines including IFN-γ that helps regulate the expression of HLA-II molecules in MSCs [25, 41]. IFN-γ binds its receptors, IFN-γR1 and IFN-γR2, to phosphorylate STAT1 and then activates the transcription of CIITA which upregulates HLA-II expression [26]. In the current study, IFN-γ stimulated the phosphorylation of STAT1 in both hiPSC-MSCs and hESC-CMs. Notably, compared with hESC-CMs, the expression of P-STAT1 was significantly reduced in hiPSC-MSCs and may lead to lower expression of HLA-II. Furthermore, we used the P-STAT1 antagonist to verify that P-STAT1 plays a critical role in regulating the expression of HLA-II. Compared with hESC-CMs, insensitivity of hiPSC-MSCs to IFN-γ stimulation results in a lower expression of HLA-II, thereby reducing the allogeneic rejection response after transplantation and ultimately enhancing the therapeutic effects. Taken together, these findings show that hiPSC-MSCs can be prepared as allogenic cells without any ethical concerns and are less susceptible to immune rejection after transplantation than hESC-CMs.

Safety evaluations including arrhythmogenesis and tumorigenesis are important concerns in cell-based therapy, as well as functional improvement. Owing to the ability of pluripotent stem cells to indefinitely proliferate and expand in an in vitro culture system, their derivatives, such as cardiomyocytes and MSCs with more stable phenotypes, offer a more feasible option. We observed no teratoma formation in either cell transplantation groups. This might be attributed to the limited long-term survival of the transplanted cells in the myocardium. On the other hand, pluripotent stem cell-derived cardiomyocytes exhibit immature electrophysiological properties compared with mature adult cardiomyocytes and are associated with a high arrhythmia risk after transplantation. We recently observed an increased incidence of spontaneous non-sustained ventricular tachyarrhythmias following hESC-CM transplantation [28], but not following hiPSC-MSC transplantation. Although we did not observe an increased risk of sudden death after transplantation, sustained ventricular tachyarrhythmias could be easily inducible in each group of animals. These results suggest that hESC-CM and hiPSC-MSC transplantation does not modify the underlying myocardial substrate to reduce susceptibility to ventricular tachyarrhythmias in HF.

## Conclusions

Our results demonstrate that transplantation of hiPSC-MSCs is safe and does not increase proarrhythmia or tumor formation and superior to hESC-CMs for improvement of cardiac function in HF via their immunomodulation to improve in vivo survival and enhance angiogenesis via paracrine effects.

### Study limitations

There are several limitations in this study. First, cell loss during the injection and immune rejection might have affected the experimental outcomes. Indeed, recent non-human primate studies showed that significant hESC-CM engraftment could be achieved with more intensity immunosuppression with steroid, cyclosporine, and abatacept in the early phase of myocardial infarction. In this study, immunosuppressive reagents used in their study (5 mg/kg/day cyclosporine + steroid) were likely to be insufficient for hESC-CMs to escape rejection. Second, the outbred pig model has limitations to examine the immunological response. Transient immunosuppressive monotherapy is usually sufficient to obtain graft survival because allograft rejection is less severe or nonexistent in inbred pigs. Unfortunately, so far, there are no inbred pigs available in HK. In our next studies, we hope to import inbred pigs, such as Wuzhishan minipig, to examine the immunological response after cell transplantation.

## Additional file

Additional file 1: Supplementary Materials and Methods. (DOCX 2775 kb)

## Abbreviations

+dP/dt: Maximal positive pressure derivative
ESPVR: End systolic pressure-volume relationship
hESC-CMs: Human embryonic stem cell-derived cardiomyocytes
HF: Heart failure
hiPSC-MSCs: Human induced pluripotent stem cell-derived mesenchymal stem cells
HLA: Human leukocyte antigen
LVEF: Left ventricular ejection fraction
MI: Myocardial infarction
VT: Ventricular tachyarrhythmia
