# Supplementary material for: Potent immunomodulation and angiogenic effects of mesenchymal stem cells versus cardiomyocytes derived from pluripotent stem cells for treatment of heart failure
Source: Stem Cell Res Ther. 2019 Mar 7;10:78. doi: 10.1186/s13287-019-1183-3 (PMC6407247; doi:10.1186/s13287-019-1183-3)
Supplement: Supplementary file 1 — Supplementary Materials and Methods. (DOCX 2775 kb) [file 13287_2019_1183_MOESM1_ESM.docx]

**Data Supplement**

**Potent Immunomodulation and Angiogenic Effects of Mesenchymal Stem Cells versus Cardiomyocytes Derived from Pluripotent Stem Cells for Treatment of Heart Failure**

Songyan Liao, MD, PhD;^1,2#^ Yuelin Zhang, MD, PhD^1,2,3#^; Sherwin Ting, BSc;^4^

Zhe Zhen, MD;^1,2^ Fan Luo, MD;^1^ Ziyi Zhu, MD;^1^ Yu Jiang;^1,2^ Sijia Sun;^1,2^ Wing-Hon Lai, PhD;^1,2^

Qizhou Lian MD, PhD^1,2,5,6*^; Hung-Fat Tse, MD, PhD.^1,2,5,6*^

^1^Cardiology Division, Department of Medicine, Queen Mary Hospital, the University of Hong Kong, Hong Kong SAR, China; ^2^ Shenzhen Institutes of Research and Innovation, the University of Hong Kong, Hong Kong SAR, China; ^3^ Department of Emergency, Guangdong Academy of Medical Sciences, Guangdong, China; ^4^Bioprocessing Technology Institute, A*STAR (Agency for Science, Technology and Research), Singapore 138668, Singapore; ^5^Research Center of Heart, Brain, Hormone and Healthy Aging, Li Ka Shing Faculty of Medicine, the University of Hong Kong**;** Hong Kong SAR, China; ^6^Hong Kong-Guangdong Joint Laboratory on Stem Cell and Regenerative Medicine, the University of Hong Kong and Guangzhou Institutes of Biomedicine and Health, China.

**Running title**: Pluripotent stem cells for heart failure

#Songyan Liao and Yuelin Zhang contributed equally to this study.

**Correspondence:**

Qizhou Lian or Hung-Fat Tse, MD, PhD

Cardiology Division, Department of Medicine,

The University of Hong Kong, Rm 1928, Block K,

Queen Mary Hospital, Hong Kong, China.

Tel: +852 22554694; Fax: +852 28186304;

Email:qzlian@hku.hk or [hftse@hkucc.hku.hk](mailto:hftse@hkucc.hku.hk)

**Supplemental Materials and Methods**

**Cell culture**

The culture of mesenchymal stem cells (MSCs) derived from human induced pluripotent stem cells (hiPSC) lines were characterized as previously described [[1-3](#_ENREF_1)]. Two hiPSC-MSC lines (Lee NL-iPSC-MSCs and IMR90-iPSC-MSCs) were cultured with DMEM plus 10% fetal calf serum (GIBCO), basic fibroblast growth factor (bFGF, 5ng/mL), and epidermal growth factor (EGF, 10ng/mL). Cells were diluted at a ratio of 1:4 when they reached confluence. The hiPSC-MSCs at passage 7~9 were used in the current study. The iPSC-MSCs were manufactured in a biosafety level two cell culture room and cryopreserved in liquid nitrogen tank in our laboratory at the Li Ka Shing Faculty of Medicine, The University of Hong Kong. Before transplantation, the cells were thawed out immediately for each animal before transplantation. As determined by trypan blue staining, the cell viability for each batch of cell was above 95% before transplantation.

The culture and differentiation of human embryonic stem cell derived-cardiomyocytes (hESC-CMs) has been described previously [[4](#_ENREF_4)]. The hESC-CMs were manufactured and differentiated at Bioprocessing Technology Institute, A*STAR (Agency for Science, Technology and Research), Singapore, and then they were transferred to HKU for further culture for 7 days before transplantation. The cells were digested by 10ug/ml collagenase IV to acquire single cell suspension immediately before transplantation for each animal. As determined by flow cytometric (FC500, Beckmen coulter) and countess cell counter analysis (Thermal Fisher), the cell viability for each batch of cell was above 95% before transplantation.

Briefly, to induce cardiac differentiation, two undifferentiated hESC lines (H-7 and HES-3) ([46 X, X]; ES Cell International) were maintained in mTeSR™1 medium (STEMCELL Technologies Inc., Vancouver, BC, Canada). CHIR99021 (CHIR) in RPMI 1640 supplemented with B27-insulin (Invitrogen) was added at a concentration of 18 μM for the first 24 h and then removed via medium change (day 0 to day 1). On day 3 of differentiation, cells were treated with 5 μM IWP-2 (Stemgent) in RPMI 1640 supplemented with B27-insulin. IWP-2 was removed via medium change on day 5 and cells were maintained in RPMI 1640 supplemented with B27-insulin thereafter. On day 12, cultures were harvested for analysis.

**Characterization of hiPSC-MSCs**

Cell surface antigens for hiPSC-MSCs were analyzed with a fluorescence-activated cell sorter (FACS).[[1](#_ENREF_1)] Cells were incubated with the following conjugated monoclonal antibodies: CD34, CD44, CD45, CD90, CD105 (R&D Systems, Minneapolis, Minn). Non-specific fluorescence was determined by incubation of similar cell aliquots with isotype-matched mouse monoclonal antibodies (BD PharMingen). Data were analyzed by collecting 20,000 events on a BD FACS Aria using FlowJo 8.8.4 software. Adipogenesis, osteogenesis, and chondrogenesis of iPSC-MSCs were carried out as previously described [[1](#_ENREF_1)]. Oil Red, Alizarin Red, and Alcian Blue were used to stain for adipocytes, osteocytes, and chondrocytes, respectively.

**Characterization of hESC-CMs**

Following our own protocol,[[4](#_ENREF_4), [5](#_ENREF_5)] the percentage of HES3-derived cardiomyocytes was quantified by fluorescence-activated cell sorter (FACS) analysis. In brief, HES3-derived cardiomyocytes were first dissociated with 1 mg/ml collagenase B (Roche Applied Sciences Penzberg, Germany). Cells were then fixed and permeabilized using a Cytofix/Cytoperm permeabilization kit (BD Biosciences, San Diego, CA). Thereafter cells were stained with monoclonal anti-troponin T antibody (cTnT; 1:200; Thermo Scientific) followed by a secondary antibody, anti-mouse IgG Phycoerythrin (PE)(dilution 1:100; Beckman Coulter, Fullerton, CA). Analysis was performed using a Beckman Coulter FC500 flow cytometer. The primary antibody, IgG_1_, was used as an isotypic control to determine background signal.

To determine whether the differentiated cardiomyocytes were ventricular-, atrial- or nodal-like, electrophysiological study by patch clamping technique on action potential was performed as previously described [[5](#_ENREF_5)]. The ratio of action potential duration at 50% repolarization (APD50) to action potential duration at 90% repolarization (APD90) of individual cells was recorded. In this study, hESC-CMs were defined as ventricular-like when the APD50:APD90 ratio was ≥0.8.

**Echocardiographic measurements**

Standard transthoracic echocardiogram including 2D and M-mode imaging was performed using a commercially available echocardiographic system (Vivid i, GE Vingmed, Horten, Norway) equipped with a 3-9 MHz transducer at baseline, 8 weeks post-MI (before cell transplantation) and 2, 4, and 8 weeks after cell transplantation. In each animal, standard 2D and M-mode echocardiograms were used to measure the left ventricular (LV) volume. To obtain the left ventricular volume using M-mode echocardiograms, the maximum minor axis of the LV at end-diastole and end-systole was measured on the parasternal long-axis view, on the assumption that the left ventricle is a spheroid at the short axis view, to calculate LV ejection fraction (LVEF) [[6](#_ENREF_6)]. All echocardiographic measurements were interpreted off-line in a blinded fashion by another independent operator using a computer workstation (GE Medical, EchoPac, Horten, Norway). The intra-observer variability of the measurement of LVEF based on M-mode measurement was 4% based on 20 repeated random measurements.

**Invasive hemodynamic assessment**

Invasive hemodynamic assessment was performed during induction of MI, before and 8 weeks after cell transplantation to assess changes in LV function as reported before [[7](#_ENREF_7)]. In brief, a 7-Fr combined catheter micromanometer (Millar Instruments, Houston, TX, USA) was calibrated in isotonic saline with a pressure–volume signal processor (CD Leycom, The Netherlands), and then advanced via the femoral artery to the LV apex to measure left ventricular maximal positive pressure derivative (LV +dP/dt) and end systolic pressure-volume relationship end-systolic pressure-volume relationship (ESPVR) during occlusion of the inferior vena cava.

**Intracardiac programmed electrical stimulation**

Programmed electrical stimulation was performed to assess the inducibility of ventricular tachyarrhythmia (VT) after cell therapy. In brief, a 6F electrophysiological catheter (Cordis Corp, Miami, FL) was inserted into the right ventricular apex via the femoral vein before sacrifice of the animals. The intracardiac recordings were displayed with the surface electrocardiogram leads I, II, and III on the CardioLab electrophysiological system (Prucka Engineering Inc., Houston, TX) at a speed of 200 mm/s. A 2-ms pulse width at twice the diastolic threshold was delivered using a stimulator (Medtronic Inc., Minneapolis, MN). After determining the right ventricular effective refractory period and the right ventricular diastolic threshold, the inducibility of VT was evaluated by programmed electrical stimulation. A pacing train of eight stimuli (S1) was delivered at two drive cycle lengths (200 and 300ms), followed by one (S2) or two (S2 and S3) premature extra stimuli. The coupling intervals were sequentially shortened until a ventricular effective refractory period or arrhythmia was induced. The presence of inducible sustained VT (>10 seconds) was noted.

**Histological examination**

The animals were sacrificed at 8 weeks after cell transplantation. Serial LV tissue samples were sectioned at 1 cm thickness in the LV transverse direction. Portions of the slices that contained infarcted myocardium were selected to measure wall thickness and infarct area. The tissue that comprised approximate 1cm^2^ pieces within, adjacent and remote to the infarct sites were embedded in paraffin, then sectioned into 5um slices for histological examination. The engraftment of the transplanted hESC-CMs was detected by immunohistochemical staining for Anti-Cardiac Troponin-T (1:200, ab64623, abcam). hiPSC-MSCs was detected by CD105 (1:200, ab11414, abcam). Polyclonal mouse anti- alpha-smooth muscle actin (α-SMA, 1:200, Sigma) was used to measure capillary density in the infarct and peri-infarct areas after cell transplantation. Regulatory T cell and macrophage expression was measured by anti-FOXP3 antibody (ab20034, abcam) and anti-macrophage antibody (MAC387, abcam) immunostaining. Quantitative analysis of positive vessels was performed in three different sections at five random fields from the peri-infarct zone in each animal. All the images of heart sections were captured by the Axio Plus image capturing system (Zeiss, GmbH, Oberkochen, Germany) and analyzed using AxioVision Rel. 4.5 software (Zeiss, GmbH). Angiogenic cytokine expression from the heart tissue at the peri-infarct site was measured by porcine cytokine array kit (RayBiotech, Norcross, GA).

**Stimulation of hiPSC-MSCs and hESC-CMs**

IFN-γ plays a critical role in regulating HLA expression in stem cells and mediates their immunomodulatory properties [8, 9]. Therefore, to mimic an inflammatory environment of infarcted heart, we used IFN-γ to stimulate hESC-CM and iPSC-MSCs and then analyzed the expression of HLA in-vitro. The hiPSC-MSCs and hESC-CMs were cultured with or without human interferon-γ (IFN-γ) (200ng/ml, GIBCO) for 24 hours and 48 hours, respectively and then the expression of Human Leukocyte Antigen Class -I (HLA-I) and Class-II (HLA-II) was examined by Western blot. Furthermore, hiPSC-MSCs and hESC-CMs were exposed to IFN-γ challenge for 0 minute, 15 minutes, 30 minutes, 60 minutes, 24hrs and 48hrs. Subsequently, Western blot was performed to detect the level of signal transducer and activator of transcription 1(P-STAT1) and total STAT1 at these time points.

**Western blot**

Western blot was performed as previously described [[10](#_ENREF_8)]. Briefly, the total protein was extracted using lysis buffer and then the concentration was measured by bicinchoninic protein assay kit. After running on 10% polyacrylamide gel, the protein was transferred to PVDF membranes. Subsequently, the membranes were incubated with the primary antibodies overnight at 4°C followed by incubation with horseradish peroxide-conjugated secondary antibody at 37°C for one hr. The primary antibodies used in this study were as follows: human HLA-I (HLA-ABC, ab20181, abcam), human HLA-II (HLA-DR, ab23755, abcam), phospho-STAT1 (Tyr701, #9167, Cell Signaling), and STAT1 (#9172, Cell Signaling).

**Conditioned medium (CdM) collection**

The CdM was prepared as previously described [[11](#_ENREF_9)]. Briefly, a total of 5 × 10^6^ hiPSC-MSCs and hESC-CMs were plated on 15-cm culture dishes. After 24 hours culture, the culture medium was aspirated and the cells were gently washed twice with phosphate buffer solution (PBS) and then 15 ml of serum-and antibiotic-free DMEM was added with or without IFN- γ. Twenty-four hours later, the supernatant was harvested gently and transferred to ultrafiltration conical tubes (Amicon Ultra-15 with membranes selective for 3kDa), and finally centrifuged (4,000 g for 30 min at 4 °C) to concentrate the CdM. The final concentration of CdM was adjusted to 20 times using DMEM and the concentration was measured by bicinchoninic protein assay kit.

**Tube formation assay in Matrigel**

To determine the angiogenetic capacity of the CdM from hiPSC-MSCs and hESC-CMs, tube formation was assessed using an in-Vitro Angiogenesis Assay Kit (Chemicon, Temecula, CA) as previously described [1[2](#_ENREF_10), 1[3](#_ENREF_11)]. Briefly, 1X10^5^ [human umbilical vein endothelial cell](https://www.google.com.hk/url?sa=t&rct=j&q=&esrc=s&source=web&cd=2&cad=rja&uact=8&ved=0ahUKEwjlluLKn6DQAhUGVZQKHdg8CxcQFgglMAE&url=https%3A%2F%2Fen.wikipedia.org%2Fwiki%2FHuman_umbilical_vein_endothelial_cell&usg=AFQjCNGIJ3Jo4L9IMrRCum5_R5bixeZNYQ&sig2=IB-EtEXctGWE94xTlUYtVg)s (HUVECs) were harvested and plated onto Matrigel-coated 24-well plates and then cultured in DMEM or CdM from hiPSC-MSCs and hESC-CMs with or without IFN- γ for 16 hours at 37°C. Tube formation was captured by microscopy, and length of tube was quantified by randomly selecting five fields per well.

**Cytokine Array**

Cytokine assay of CdM from hiPSC-MSCs and hESC-CMs was assessed by RayBio® Cytokine antibody array as previously described [[9](#_ENREF_9)]. Briefly, a total of 200μl CdM from hiPSC-MSCs and hESC-CMs with or without IFN-γ stimulation was assayed according to the manufacturer’s instructions (RayBiotech, Norcross, GA). The cytokines were quantified using Quantibody Q-Analyzer.

**Supplemental Table Legend**

**Table S1. Echocardiographic measurement after MI and cell transplantation.** Left ventricular end systolic diameter (LVESD) was significantly decreased in hiPSC-MSCs group at 4 and 8 weeks after transplantation compared with during HF. But left ventricular end diastolic diameter (LVEDD), left atrial dimension (LAD) and the ratio of peak velocity flow in early diastole to late diastole (E/A ratio) has no significant difference among all groups.

**Supplemental Figure Legends**

**Figure S1.Charaterization of human iPSC-MSCs. (A).** Surface antigen profiling of iPSC-MSCs for CD34, CD44, CD45, CD90 and CD105 with fluorescence activated cell sorter (FACS) analysis. The cultured MSCs expressed on their surface marker CD44, CD90 and CD105, but lacked expression of CD34 and CD45. (**B).** Differentiation capacity of iPSC-MSCs. Oil red O staining for adipogenesis; Alizarin red staining for osteogenesis; Alcian blue staining for chondrogenesis.

**Figure S2. Fluorescence activated cell sorter (FACS) analysis of cardiac-specific markers.** Percentage of troponin-T positive hESC-CMs was determined to be 80±4% (n=4) by flow cytometry.

**Figure S3. Cellular electrophysiological properties. (A).** Single cell action potential waveforms of atrial-, nodal- and ventricular-like phenotypes recorded by patch clamping technique. **(B).** The percentage of ventricular-like cardiomyocytes is 63±6% (n=4) in the hESC-CMs.

**Figure S4. Porcine model of myocardial infarction.**  Porcine model of myocardial infarction (MI) was induced by left circumflex (LCX, red arrow) coronary artery embolization. The obtuse marginal branch of the LCX was occluded with balloon inflation and 700 mm microspheres were injected to generate MI. Coronary angiography at pre-MI, balloon inflation and post-MI was performed through a 6F JR4 guiding catheter via the right femoral artery.

**Figure S5. Cell transplantation in a porcine model of MI. (A).** The morphology of hiPSC-MSCs and hESC-CMs in culture. (**B).** Cell injection sites at the lateral wall around the infarct area of left ventricle during left thoracotomy. The blue arrow shows the peri-infarct area and the red arrow shows the infarct area.

**Figure S6.**  **Arrhythmogenic complications after cell transplantation. (A).** The incidences of ventricular tachyarrhythmias (VT, red arrow) induced by *in-vivo* intracardiac programmed electrical stimulation. (**B).** The incidence of spontaneous non-sustained VT was higher in the hESC-CM group (n=8) than hiPSC-MSC and MI group (n=8). **(C).** The incidence of inducible sustained VT did not differ significantly between the three experimental groups.

**Figure S7. No any tumor formation at the injection areas as well as the other sites over the myocardium or other organs**

**Figure S8**. **Number of native cardiomyocytes and area are significantly different between the hESC-CMs and hiPSC-MSCs groups at the peri-infacrt area.** H&E staining of heart tissues at infarct area (A) and prei-infarct area (B) 8 weeks after transplantation. Myocyte area(C,E) and number (D,F) in the hESC-CMs and hiPSC-MSCs groups has significantly difference compared to MI group in both infarct and peri-infacrt area. Moreover, Myocyte area (E) and number(F) has significantly difference between the hESC-CMs and hiPSC-MSCs groups at the peri-infacrt area.

**Table S1. Transthoracic echocardiographic parameters**

| **Parameters** | **MI (n=8)** | **hESC-CM (n=8)** | **hiPSC-MSCs (n=8)** |
| --- | --- | --- | --- |
| **Left ventricular end-diastolic dimension (cm)** | | | |
| Baseline  Heart failure  Transplantation 2w  Transplantation 4w Transplantation 8w | 3.5±0.5  4.3±0.5  4.3±0.4  4.5±0.3  4.4±0.5 | 3.7±0.4  4.8±0.6  4.9±0.4  4.9±0.4  4.7±0.5 | 3.8±0.4  5.0±0.4  4.9±0.5  4.8±0.3  4.5±0.4 |
| **Left ventricular end-systolic dimension (cm)** | | | |
| Baseline  Heart failure  Transplantation 2w  Transplantation 4w Transplantation 8w | 2.3±0.5  3.6±0.5  3.5±0.4  3.6±0.3  3.6±0.4 | 2.5±0.3  3.6±0.5  3.6±0.4  3.6±0.4  3.4±0.5 | 2.5±0.3  4.0±0.4  3.7±0.4  3.5±0.4*  3.3±0.3* |
| **Left atrial dimesion (cm)** | | | |
| Baseline  Heart failure  Transplantation 2w  Transplantation 4w Transplantation 8w | 2.7±0.5  3.4±0.3  3.5±0.4  3.3±0.4  3.4±0.2 | 2.5±0.5  3.3±0.3  3.2±0.4  3.3±0.3  3.1±0.3 | 2.7±0.4  3.5±0.7  3.5±0.5  3.2±0.3  3.3±0.4 |
| **Mitral Doppler flow E/A ratio** | | | |
| Baseline  Heart failure  Transplantation 2w  Transplantation 4w Transplantation 8w | 1.3±0.1  1.2±0.2  1.1±0.2  1.2±0.1  1.1±0.1 | 1.3±0.3  1.2±0.3  1.3±0.1  1.3±0.2  1.2±0.1 | 1.3±0.1  1.2±0.2  1.3±0.2  1.3±0.2  1.3±0.1 |

*p<0.05 vs. HF

**Supplemental Figure 1**


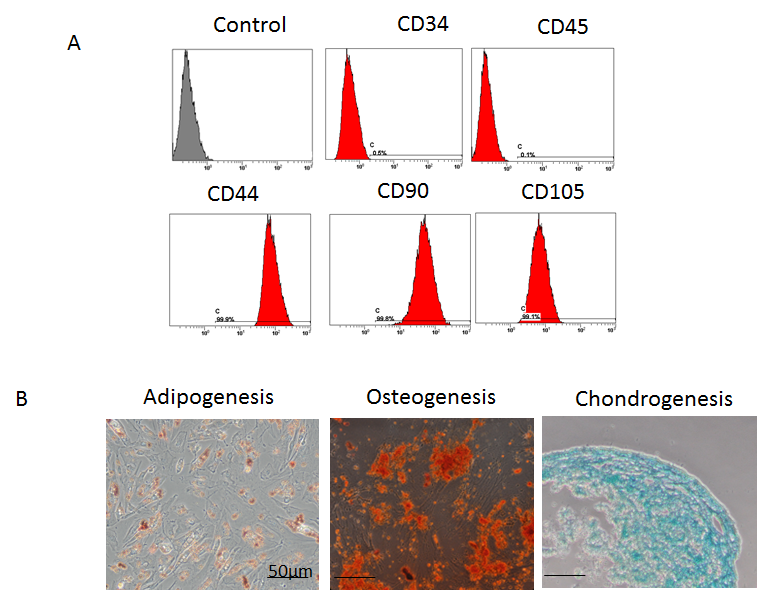


**Supplemental Figure 2
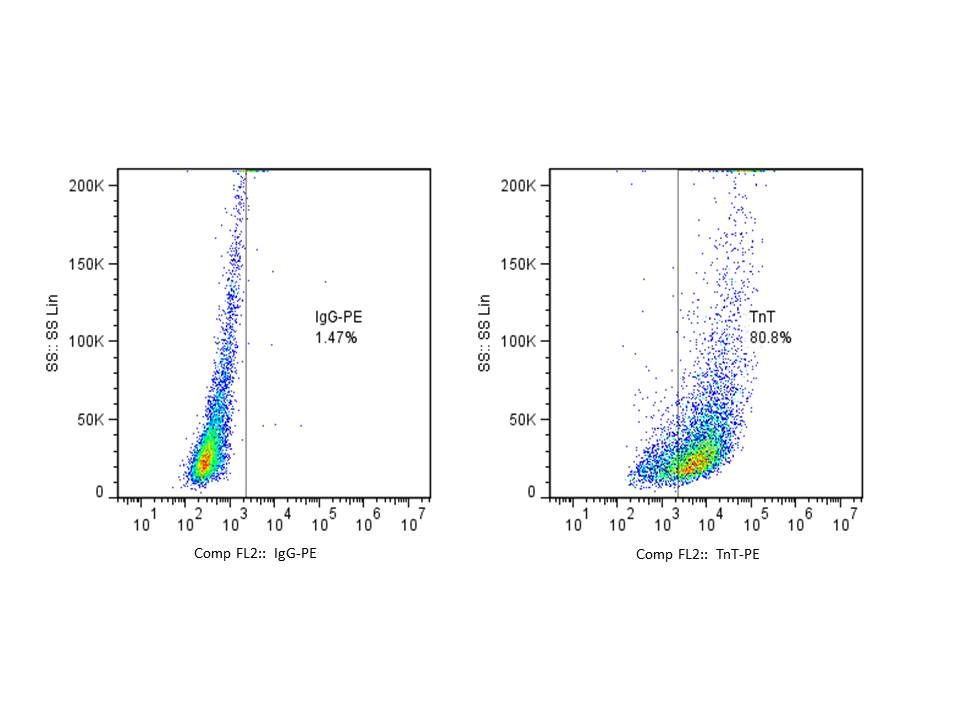
**

**Supplemental Figure 3**

**
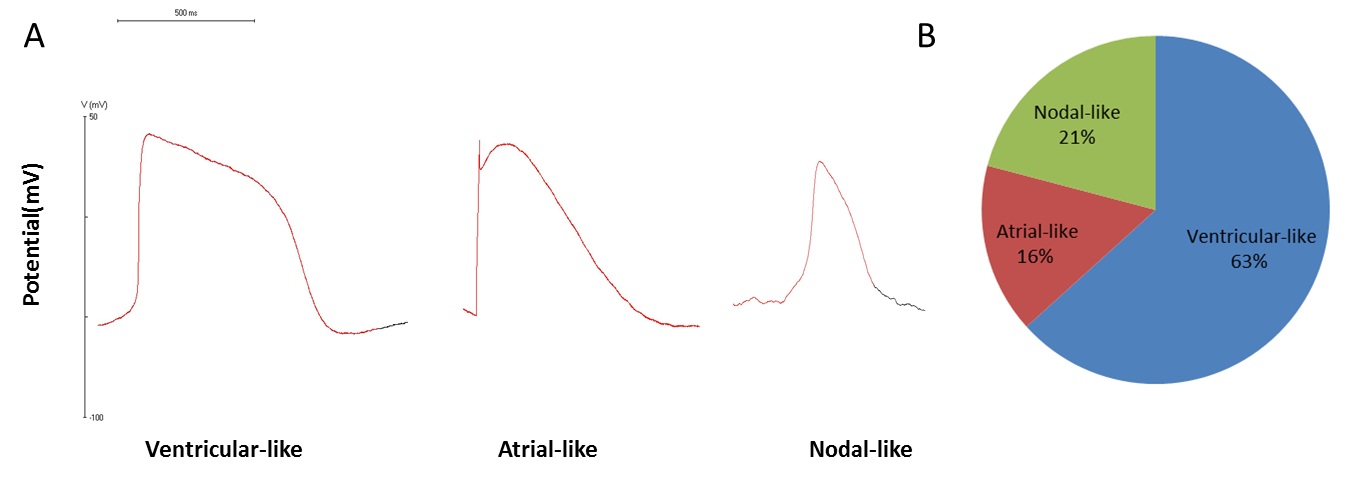
**


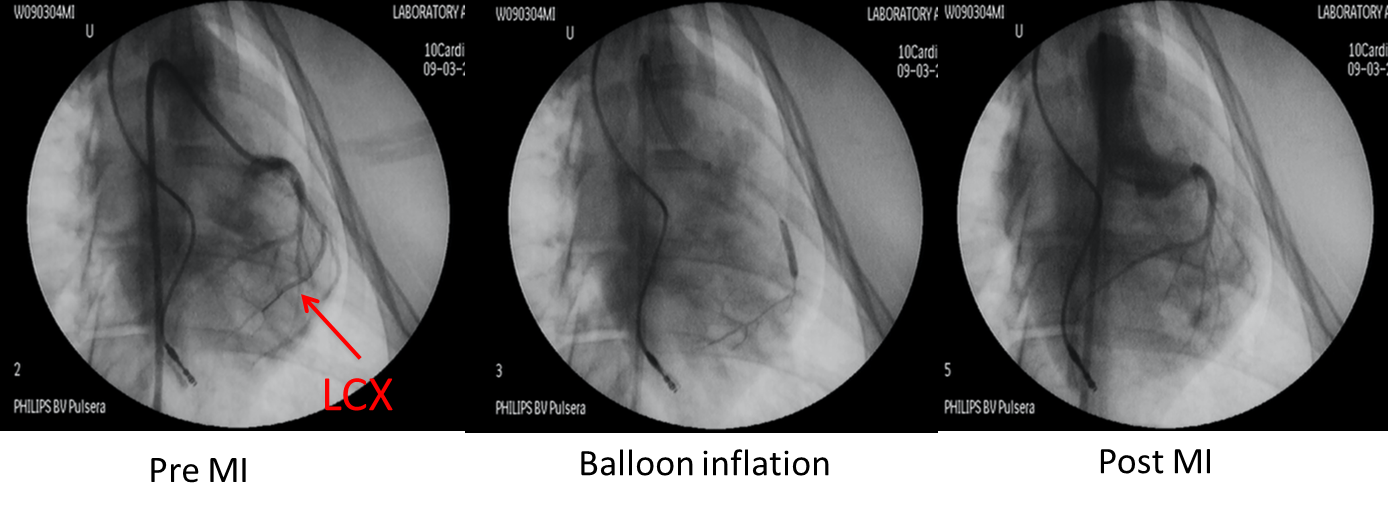
**Supplemental Figure 4**

**Supplemental Figure**
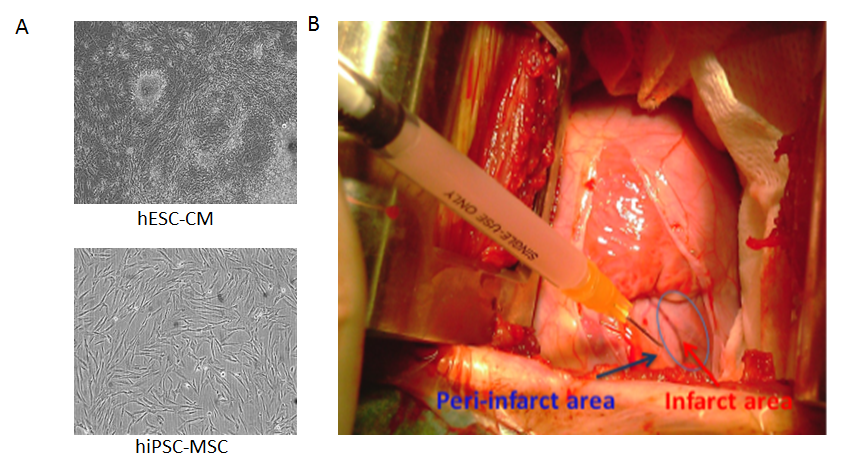
**5**

**Supplemental Figure 6**

**A**


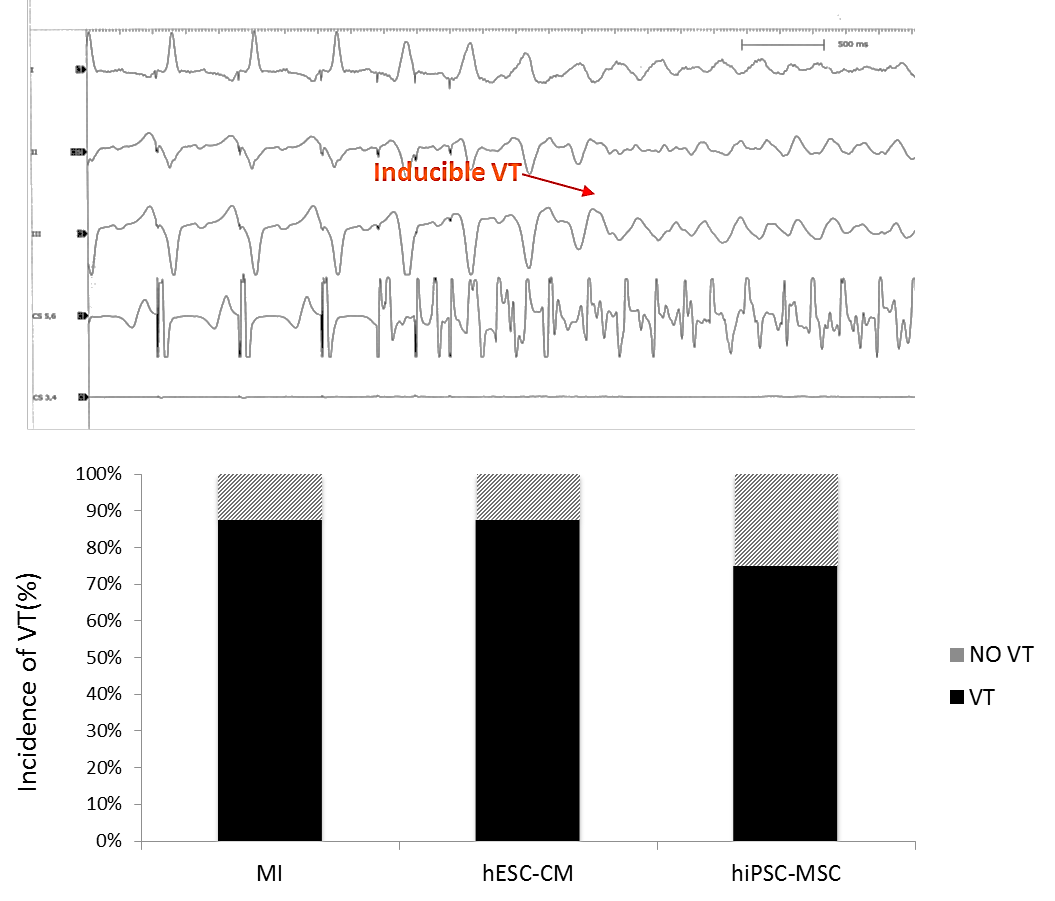


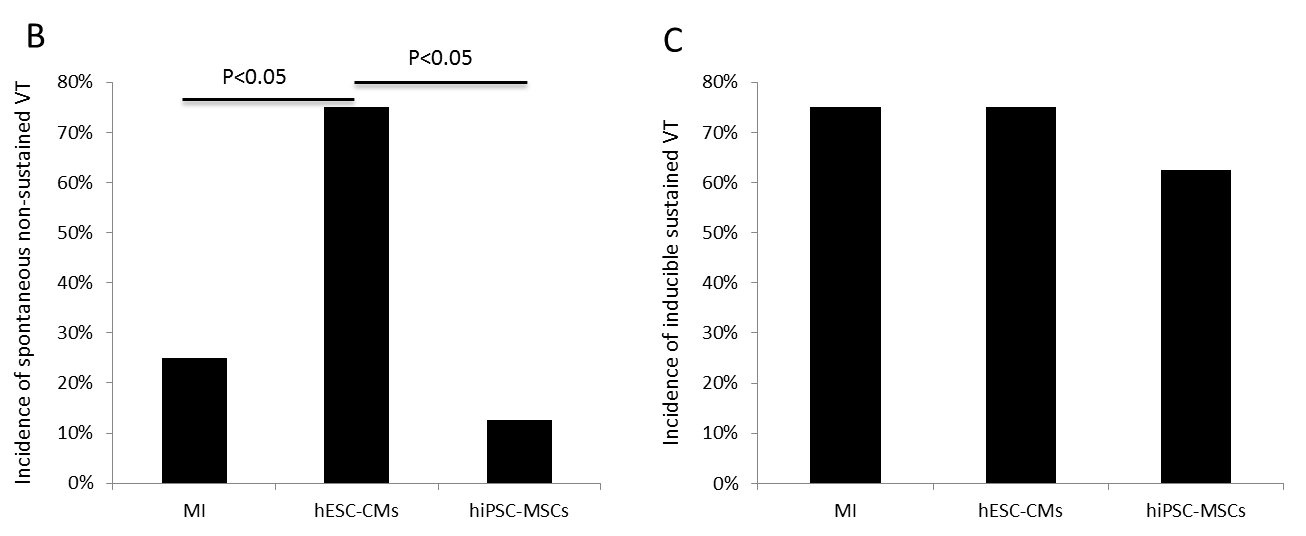


**Supplemental Figure 7**


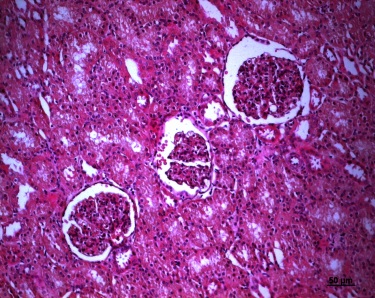

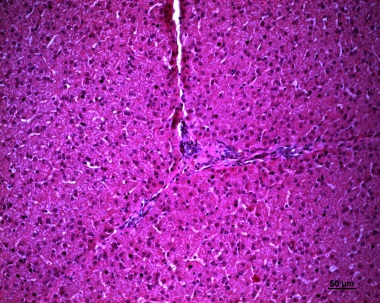

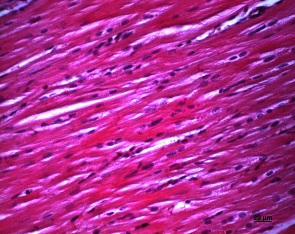


**Kidney Liver Left Ventricle**


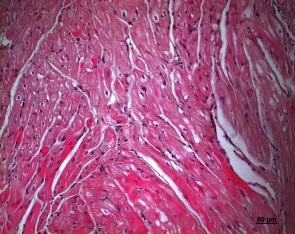

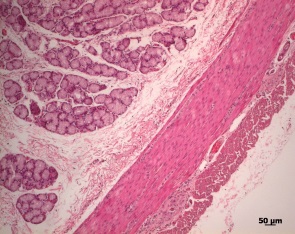

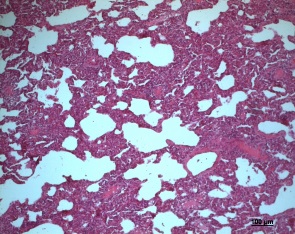


**Right Atrium Duodenum Lung**

**Supplemental Figure 8.**


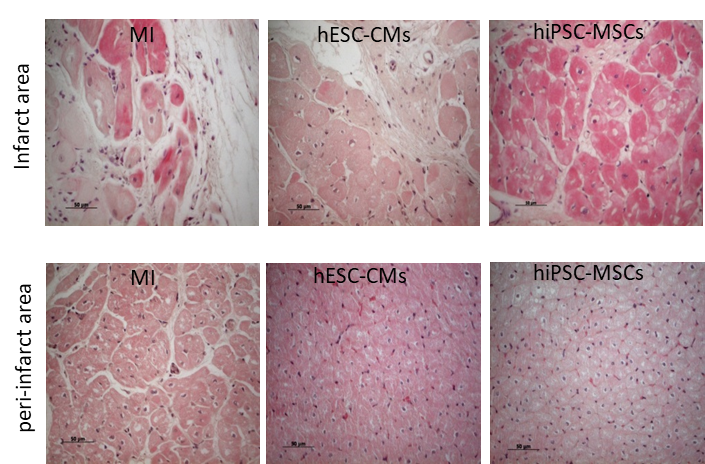


B

A


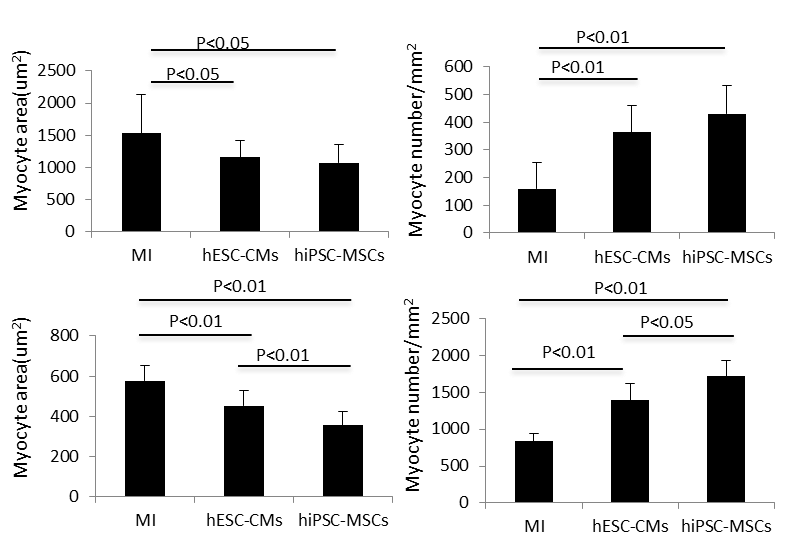


D

C

E

F

**Reference:**

1. Lian Q, Zhang Y, Zhang J et al. Functional mesenchymal stem cells derived from human induced pluripotent stem cells attenuate limb ischemia in mice. Circulation 2010; 121: 1113-23.

2. Zhang J, Lian Q, Zhu G et al. A human iPSC model of Hutchinson Gilford Progeria reveals vascular smooth muscle and mesenchymal stem cell defects. Cell Stem Cell 2011; 8: 31-45.

3. Lian Q, Zhang Y, Liang X et al. Directed Differentiation of Human-Induced Pluripotent Stem Cells to Mesenchymal Stem Cells. Methods Mol Biol 2016; 1416: 289-98.

4. Ting S, Chen A, Reuveny S, Oh S. An intermittent rocking platform for integrated expansion and differentiation of human pluripotent stem cells to cardiomyocytes in suspended microcarrier cultures. Stem Cell Res 2014; 13: 202-13.

5. Chan YC, Ting S, Lee YK et al. Electrical stimulation promotes maturation of cardiomyocytes derived from human embryonic stem cells. J Cardiovasc Transl Res 2013; 6: 989-99.

6. Lopshire JC, Zhou X, Dusa C et al. Spinal cord stimulation improves ventricular function and reduces ventricular arrhythmias in a canine postinfarction heart failure model. Circulation 2009; 120: 286-94.

7. Liao SY, Liu Y, Zuo M et al. Remodelling of cardiac sympathetic re-innervation with thoracic spinal cord stimulation improves left ventricular function in a porcine model of heart failure. Europace 2015; 17: 1875-83.

8. Sun YQ, Zhang Y, Li X, et al. Insensitivity of Human iPS Cells-Derived Mesenchymal Stem Cells to Interferon-γ-induced HLA Expression Potentiates Repair Efficiency of Hind Limb Ischemia in Immune Humanized NOD Scid Gamma Mice. Stem Cells. 2015 Dec;33(12):3452-67

9. Fu X, Chen Y, Xie FN, et al. [Comparison of immunological characteristics of mesenchymal stem cells derived from human embryonic stem cells and bone marrow.](https://www.ncbi.nlm.nih.gov/pubmed/25256849) Tissue Eng Part A. 2015 Feb;21(3-4):616-26

10. Zhang Y, Yu Z, Jiang D et al. iPSC-MSCs with High Intrinsic MIRO1 and Sensitivity to TNF-alpha Yield Efficacious Mitochondrial Transfer to Rescue Anthracycline-Induced Cardiomyopathy. Stem Cell Reports 2016; 7: 749-63.

11. Li X, Zhang Y, Yeung SC et al. Mitochondrial transfer of induced pluripotent stem cell-derived mesenchymal stem cells to airway epithelial cells attenuates cigarette smoke-induced damage. Am J Respir Cell Mol Biol 2014; 51: 455-65.

12. Lai WH, Ho JC, Chan YC et al. Attenuation of hind-limb ischemia in mice with endothelial-like cells derived from different sources of human stem cells. PLoS One 2013; 8: e57876.

13. Liang X, Zhang L, Wang S et al. Exosomes secreted by mesenchymal stem cells promote endothelial cell angiogenesis by transferring miR-125a. J Cell Sci 2016; 129: 2182-9.
